# Bathymetric data for Israeli coastal micro-estuaries

**DOI:** 10.1016/j.dib.2023.109444

**Published:** 2023-07-26

**Authors:** Yair Suari, Dror Suari, Yonatan Suari, Tal Sade, Hadar Sedaka, Tom Topaz

**Affiliations:** a Faculty of Marine Science, Ruppin Academic Center, Michmoret 40297, Israel; b Hadera Democratic School, Horshat Brandeis, Hadera 38242, Israel

**Keywords:** Mediterranean, Streams, Brackish, Lagoons

## Abstract

Data from a bathymetric mapping project conducted in seven Israeli coastal micro-estuaries (Lachish, Sorek, Yarkon, Alexander, Hadera, Taninim, and Kishon) is presented. The data were collected by rowing a kayak along an S-shaped track through the estuaries. An echosounder equipped with a Global Positioning System (GPS) unit were mounted on the kayak. The data preparation consisted of a) manual removal of outliers, mostly caused by instrument echo in water depths below the instrument's 0.5 m minimum; b) correction of the measured water level to sea level; and c) interpolation of the sampling points into a regular grid using a terrain-following interpolation algorithm. For each of the estuaries, the raw measurements as a text (csv) file and the interpolated data both as a text (CSV) file and a GeoTiff file were produced.

**Specifications Table**

**Table:** 

Subject	Earth and Planetary Sciences, Geophysics
Specific subject area	Bottom depths of coastal micro-estuaries in Israel
Type of data	Table Image (geolocated)
How the data were acquired	The data were acquired in a series of sampling days from 2015 to 2023. Sampling was conducted using a single beam echo-sounder towed behind a Kayak in S shaped tracks between the estuary banks. The depth measurements were corrected to mean sea level using an RTK-DGPS.
Data format	Raw Analyzed
Description of data collection	Bathymetric data were collected using a hummingbird Helix 10 echosounder (fish finder) mounted on a float and towed from a kayak (Fig. 1). Positions were determined using the fish finder's internal GPS unit, and the water level was corrected to mean sea level by continuously measuring the estuarine water level compared to reference points that were measured using RTK- differential GPS. Data points were logged in 1m interval along an S shaped track reaching the banks roughly once every 30m. Data validation was conducted by manually inspecting adjacent sampling points and removing noncontinuous depths, less than 1/1000 samples were removed.
Data source location	Ruppin Academic Center, Faculty of Marine Science, Michmoret, Israel All data points were geolocated in Israel, and each data point was individually geolocated in both EPSG 4326 (wgs84) and EPSG 2039 (Israeli ITM grid).
Data accessibility	Repository name: PANGEA [1] Data identification number: 10.1594/PANGAEA.959008 Direct URL to data: https://doi.pangaea.de/10.1594/PANGAEA.959008

## 1 Value of the Data


 

• These are the first bathymetric measurements in the Israeli coastal streams.
• The data enables to calculate the micro-estuaries volume, surface area and residence time [2].
• The data can be used for future assessments of changes in riverbed.
• The bottom profile can be used to identify regions of the estuaries that are likely exposed to extreme anoxic conditions [3].
• The bathymetric data can be used as input for hydrodynamic modelling [4]

## 2 Objective

The objective of the Israeli Estuarine Research Center is to acquire scientific insights about the estuaries in Israel and worldwide. Because the bathymetry of estuaries is an important determinant of the functioning of those ecosystems, we produced this dataset in the hopes that it will be used to boost further estuarine research.

## 3 Data Description

**Fig. 1:**
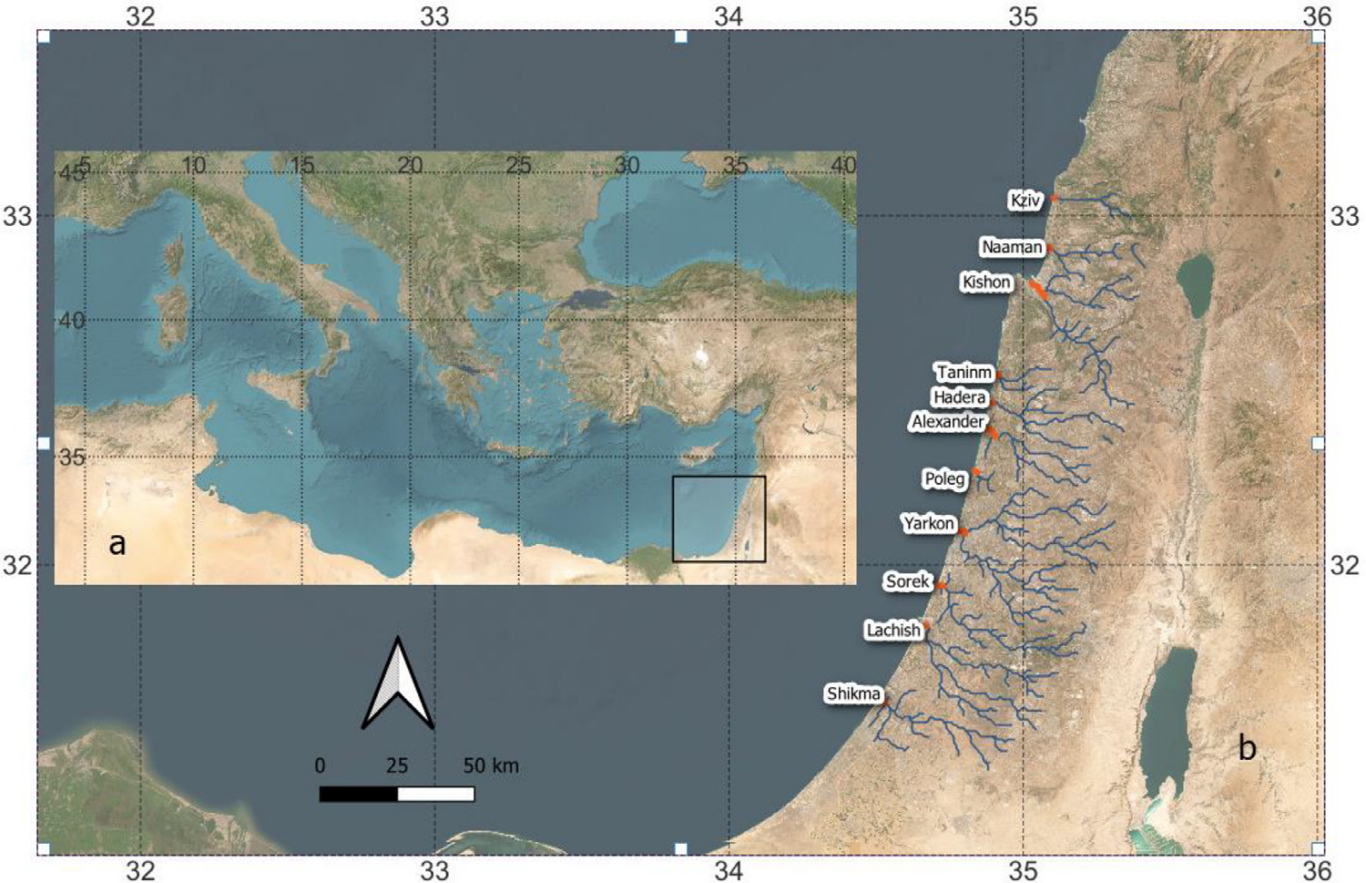
The Israeli micro-estuaries sampled in this study. a: The rectangle illustrates the location of the Israeli coastline in the eastern Mediterranean that apears in b. b: The Israeli coastal streams (in blue) and their micro-estuaries (orange). The white arrow points north and the grids apear in geographic coordinate system (degrees east and north).

We created a bathymetric dataset of seven Israeli coastal micro-estuaries (Lachish, Sorek, Yarkon, Alexander, Hadera, Taninim, and Kishon) (Table 1, Fig. 1). Naaman was not sampled due to low water quality, and Kziv, Poleg, and Shikma were mostly too shallow to sample. For each of the sampled micro-estuaries, the dataset included the raw samples (after data validation) in a CSV text file and the interpolation product in 1 m spatial resolution for the full surface area. These datasets are stored in the PANGEA repository A complete description of the files and the fields in each file are provided in a file named Field_List.xlsx that is contained in the repository.

**Table 1:** Sampled estuary's locations.

Estuary	Lon	Lat
Alexander	34.8836	32.3873
Taninim	34.8942	32.5425
Lachish	34.6446	31.8176
Kishon	35.0337	32.8184
Sorek	34.7102	31.9415
Yarkon-Ayalon	34.7800	32.1047

## 4 Experimental Design, Materials and Methods

**Fig. 2:**
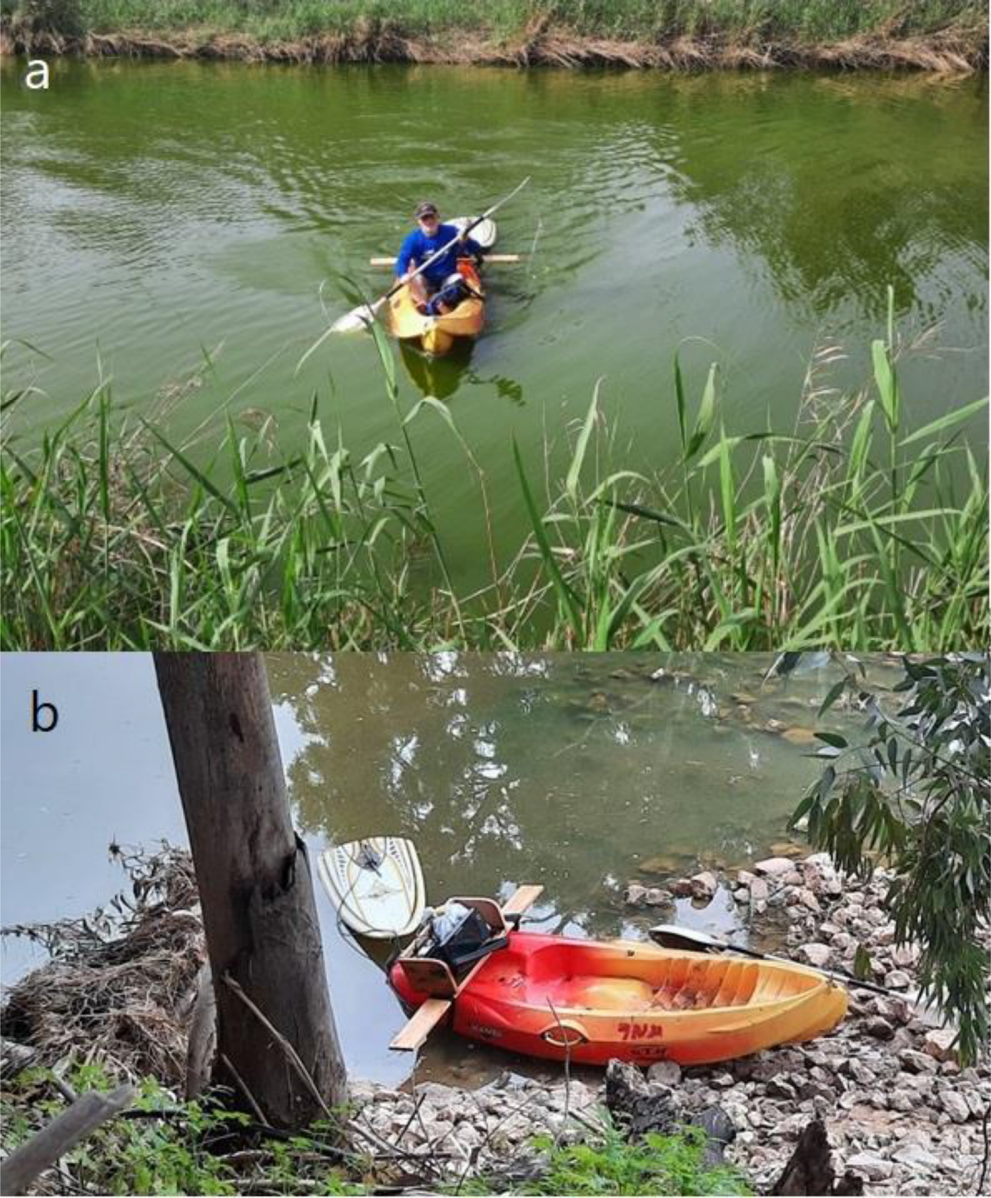
a) Sampling in the Lachish estuary – padling the Kayak. b) The sampling gear, an echosounder mounted on a Kayak, while the probe is towed behind at the surface.

**Fig. 3:**
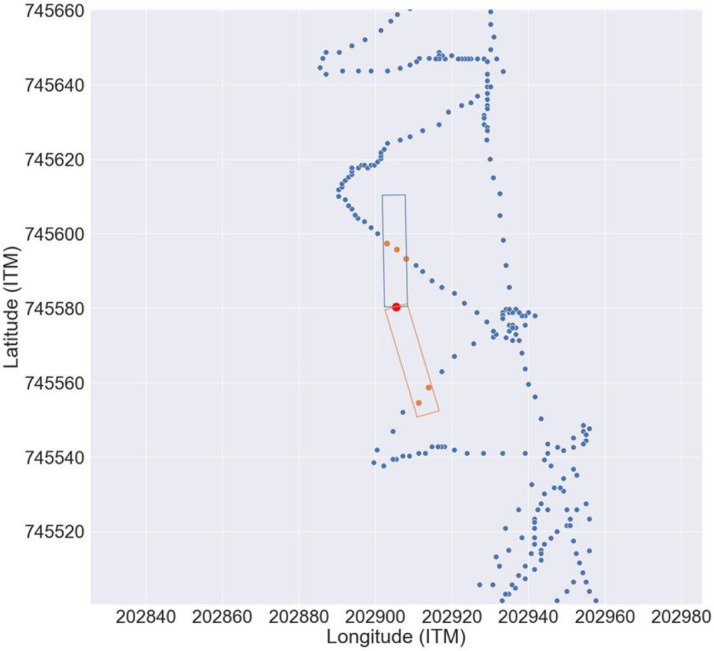
Sampling points (blue), one interpolation grid point (red), and the selection of data sampling points for interpolation (orange) at a section near a curve of the Kishon Estuary. The blue points indicate the S-shaped track that was followed during sampling. The red point is one point from the output grid of the bathymetry, and the sampling points that were used to calculate the depth of the output point were selected using the two rectangles that are tilted based on the estuary orientation upstream and downstream from the product grid point. The map is projected on the ITM (EPSG 2039) coordinate reference system.

Sampling was conducted in three separate projects that were conducted during the years 2015-2023 (Table 2) using a Hummingbird Helix 10 echosounder (fish finder) mounted on a sit-on-top kayak (Fig. 2a). This echosounder was towed near the kayak (Fig. 2b). The sampling was performed by rowing in an S-shaped track between the estuary banks (Figs. 2a, 3), and the temporal sampling resolution was 1 Hz.

**Table 2:** Sampling timing and duration for each of the estuaries.

Stream	Number of sampling days	Sampling timing
Alexander	5	April-June 2015
Taninim	1	August 2015
Lachish	3	April 2021
Kishon	2	May 2021
Sorek	1	June 2021
Yarkon-Ayalon	2	April 2023

Before sampling, an elevation reference point was marked at the mid-estuary, and the vertical distance between this point and the water level was measured once per hour. After sampling all the estuaries (in each project), the elevation of the reference points relative to the Israeli Land Survey Vertical Datum was calculated using a TOPCON GR-3 RTK-DGPS system.

Data validation was conducted by manually inspecting the depth of each of the sampling points compared to the surrounding points. The most common sampling error by far was recordings of the signal echo as depth in shallow water, which was easy to identify because it indicates deep water near the banks. Therefore, these points and shallow data points in the mid-estuary were manually removed. Generally, less than 1 point in 1000 samples in each of the estuaries were removed.

Due to shallow water, complex bathymetry, and many disturbances, sampling using a motorized vessel or a multibeam echosounder was not feasible. Therefore, sampling was conducted by kayak, and the density of data points was lower than is customary in bathymetric sampling. After careful consideration, a terrain following interpolation algorithm was selected. The interpolation of the sampled data to a regular grid was conducted by transforming the location of the sampled point from the metric Israel transverse Mercator coordinate system (ITM, EPSG 2039) into a terrain-following coordinate system where sampling positions are transformed from northing and easting to distance from outfall and distance from the estuary centerline.

Before running the interpolation code, estuary boundaries polygon and estuary centerline were manually digitized using QGIS. The centerline was represented as points with 1 m between points.

The terrain-following interpolation (Fig. 3) converted the north-south and west-east coordinates into longitudinal distance from the estuary outfall (l) and widthitudinal distance from the centerline (w). “l” was determined by finding the nearest centerline point for each sampling point and relating the centerline point l value to the sampling point. The widthitudinal distance from centerline w was then calculated as the distance between the sampling point and the centerline point.

After calculating l and w for each sampling point, the code looped the output grid points. For each output point, it selected the sampling points that were located within two rectangles, one upstream and one downstream from the output grid point (Fig. 3). These rectangles were aligned according to the mean alignment of the centerline upstream and downstream from the output grid point. Next, every selected sampling point was assigned a weight according to Eq. (1):

$$Weight=\sum_{q=1}^{q=q_{max}}\sqrt{\Delta w^{2}+\Delta l}$$

where Δw is the distance between the sampling point and the interpolation grid point along the width axis, Δl is the distance along the length axis, q is the index of the array of selected points (Orange points in In Fig. 3), and $q_{max}$ is the number of points in the two rectangles.

After calculating the weight of all selected points, the interpolated depth was calculated according to Eq. (2) where n is the index of the interpolated grid point:

$$Depth_{n}=\frac{\sum_{q=1}^{q=q_{max}}Depth_{q}*Weight_{q}}{\sum_{q=1}^{q=q_{max}}Weight_{q}}$$

## Ethics Statement

The authors declare that their work meets the requirements for publication in data in brief.

## CRediT authorship contribution statement

**Yair Suari:** Investigation, Software. **Dror Suari:** Investigation. **Yonatan Suari:** Validation, Software. **Tal Sade:** Project administration. **Hadar Sedaka:** Supervision. **Tom Topaz:** Funding acquisition.

## Data Availability

Single beam bathymetry processed data (Humminbird Helix 10 echosounder entire dataset), Israeli coastal estuaries Lachish, Sorek, Yarkon, Alexander, Taninim and Kishon. PANGAEA, (Original data) (PANGAEA).
